# Breeding of Early Maturity, High-Quality *Auricularia heimuer* Strains Using Protoplast Fusion

**DOI:** 10.3390/jof12080591

**Published:** 2026-08-10

**Authors:** Xin Yue, Shuyuan Song, Xiaojia Zhang, Yanshu Zhao, Yuxin Jiang, Shuyang Zhou, Xiancheng Zhang, Chuang Han, Xiaodong Dai

**Affiliations:** Institute of Microbiology, Heilongjiang Academy of Sciences/National Collection of Edible Fungi (Heilongjiang), Harbin 150010, China; y13474850905@163.com (X.Y.); songshuyuanssy@126.com (S.S.); zhangxiaojia1998@163.com (X.Z.); zhaoyanshu0213@163.com (Y.Z.); xs62929@163.com (Y.J.); zhoushuyang1983@163.com (S.Z.); zhangxiancheng@yeah.net (X.Z.)

**Keywords:** *Auricularia heimuer*, protoplast fusion, agronomic trait evaluation, transcriptome

## Abstract

*Auricularia heimuer* is an important cultivated edible mushroom in China. This study used the early maturity, chrysanthemum-shaped, high yield strain “8808” and the late-maturity, single-shaped, high-quality strain “HW15” as parental strains. The objective was to develop a new strain of *A. heimuer* combining early maturity with excellent quality through protoplast fusion. Protoplasts from the parental strains were fused using a polyethylene glycol (PEG)-mediated method. The obtained hybrids were identified through clamp connection, antagonism tests, esterase isozyme analysis, and Inter-Simple Sequence Repeat (ISSR) molecular marker techniques, followed by ultimate selection through cultivation. Among the evaluated hybrids, strain R33 exhibited desirable agronomic traits: primordium formation occurred 4 days earlier than the parental strain HW15, harvest time was advanced by 6 days, yield increased by 14.7%, and the ratio of absorbed water improved by 29.8%. Transcriptomic analysis revealed that starch and sucrose metabolism as well as amino acid metabolism pathways were significantly upregulated in R33, which may be associated with its early maturity and high-yield phenotype. Overall, protoplast fusion technology can be used to create promising candidate strains that combine early maturity, high yield, and excellent quality.

## 1. Introduction

*Auricularia heimuer* F. Wu, B. K. Cui & Y. C. Dai is the second most extensively cultivated edible mushroom in China [1]. It possesses both edible and medicinal value, with benefits including antitumor, antioxidant, hypoglycemic, and lipid-lowering effects [2,3,4,5]. According to statistics from the China Edible Fungi Association, the nationwide production of *A. heimuer* reached 7.1341 million tons in 2024 [6]. With the rapid development of the edible mushroom industry, market demands for both the yield and quality of *A. heimuer* have been increasing continuously. Breeding new strains with desirable traits such as early maturity and high yield can significantly shorten the cultivation cycle and reduce production costs, which has become a key objective of current breeding efforts.

Currently, the predominant strategies employed in edible mushroom breeding include natural breeding, hybrid breeding, and mutagenesis breeding. However, these approaches often face challenges such as lengthy breeding cycles and low hybrid compatibility. Protoplast fusion breeding offers a means to overcome hybridization incompatibility barriers, thereby enabling the recombination and exchange of genetic material [7]. This technology has achieved notable success in breeding research of various edible mushroom, including *Ganoderma lucidum* [8], *Morchella esculenta* [9], *Pleurotus sajor-caju* [10], and *Volvariella volvacea* [11]. Nevertheless, research on *A. heimuer* remains relatively limited.

Existing reports have primarily focused on technical aspects such as the preparation of protoplasts and the optimization of regeneration conditions [12], as well as the establishment of fusion systems [13]. However, research on directed fusion breeding guided by target traits remains scarce. At the same time, the screening of hybrids relies heavily on phenotypic observations and lacks a stable, efficient genetic marker-assisted screening system, resulting in a lengthy screening process. Furthermore, there is a lack of systematic and in-depth research on the molecular mechanisms underlying the genetic stability of hybrids, the patterns of trait segregation in offspring, and the formation of hybrid superiority. This has hindered the precise breeding and industrial application of superior fusion strains. Therefore, two parental strains with complementary characteristics were selected in this study: one exhibiting early maturity and high yield, and the other characterized by late maturity and superior quality. Protoplast fusion technology was subsequently employed to achieve the targeted integration of desirable traits, specifically combining early maturity, high yield, and premium quality. This solves the industry challenge of balancing growth rate and quality in existing varieties. This has significant theoretical and practical implications for broadening the genetic base of *A. heimuer* and accelerating the breeding of new varieties. It is necessary to establish an efficient technical system for protoplast fusion of *A. heimuer*. Furthermore, a multi-tiered, progressive identification system for hybrids was constructed, integrating morphological observation, biochemical assays, molecular marker analysis, and cultivation trials. Transcriptome sequencing is a key tool for analyzing the mechanisms of gene expression regulation. It enables the rapid identification of differentially expressed genes and key pathways associated with early maturity traits. This provides precise technical support for the molecular analysis of early maturity traits. Previous studies have identified key genes associated with the growth and development of *A. heimuer* through transcriptomic sequencing. However, there have been few reports on the specific pathways and core regulatory genes involved in early maturity traits [14,15].

This study used the early maturity, chrysanthemum-shaped, high-yield strain “8808” and the late-maturity, single-shaped, high-quality strain “HW15” as the parental strains. The 8808 strain is a traditional *A. heimuer* cultivar in Northeast China. It was bred in 1988 and was recognized as a variety by both the national and Heilongjiang Province. It has the characteristics of early maturity and high yield. And it has been used in production for over 30 years. Due to the poor morphology of the fruiting bodies, which does not meet the current market demands, it has gradually been withdrawn from production [16]. “HW15” is currently in use, single-shaped, and has a high quality. It was bred in 2015 and obtained a patent for the strain as well as a provincial-level variety recognition from Heilongjiang Province. However, this variety has a relatively late maturity and a long growth cycle [17]. The 8808 strain is a typical early maturity and high-yield variety. HW15 is characterized by its late maturity and high quality. It is currently one of the main varieties cultivated. The core economic traits of the two are complementary and precisely address the shortcomings of existing varieties in the industry. Their breeding periods span a wide range of time and their genetic backgrounds differ significantly. Following protoplast fusion, there is extensive nuclear–cytoplasmic recombination, making it easier to achieve trait segregation and produce superior hybrid strains. The genetic distances between the two are significant, enabling efficient elimination of false hybrids. This has enhanced the feasibility and accuracy of the entire experimental identification system. Using microscopy observation, antagonistic test, esterase isoenzyme analysis, and Inter-Simple Sequence Repeat (ISSR) molecular-marker techniques, the hybrid R33 was comprehensively characterized from multiple aspects, including morphology, physiology, biochemistry, and molecular biology. This step-by-step verification strategy ensures the accuracy and reliability of the results. At the same time, transcriptome sequencing was employed to identify the metabolic pathways and key genes potentially associated with early maturity. This is of considerable significance for advancing the high-quality development of the *A. heimuer* industry.

## 2. Materials and Methods

### 2.1. Strains and Media

The *A. heimuer* strains “8808” (HMCC50024) and “HW15” (CGMCC8553) were both provided by the National Edible Mushroom Germplasm Resource Bank (Heilongjiang). The required media component is shown (Table S1).

### 2.2. Preparation and Identification of Protoplasts

The preparation and regeneration of protoplasts were performed according to the method described by Han et al. [18]. Identification methods [19]: after adding fluorescent nuclear dyes to the candidate strains, the nuclear phases of the hyphae were observed under a fluorescence microscope. Through antagonism tests, different nuclear types of monokaryotic strains were identified. The esterase isozyme of the test strain was analyzed by vertical plate polyacrylamide gel electrophoresis (PAGE). Mycelial growth rate was measured by the plate method using cPDA (complete Potato Dextrose Agar) medium.

Mycelial growth rate/(cm·d^−1^) = Increase in the diameter of the mycelial growth ring/Incubation time

### 2.3. Fusion of Protoplasts

When the concentration of the protoplasts of the parental strain was 1 × 10^6^ cells/mL, they were respectively subjected to heat inactivation and ultraviolet (UV) inactivation, and their lethal values were determined. Protoplast suspensions of equal concentration and volume, each subjected to a distinct inactivation method, were mixed into a single centrifuge tube. After low-speed centrifugation, the supernatant was removed and the pellet was resuspended in 30% PEG 4000, followed by incubation at 32 °C for 30 min in a water bath. The treated protoplasts were then washed and centrifuged twice with 0.5 mol/L sucrose as the osmotic stabilizer. A total of 100 μL of protoplast suspension was pipetted onto the regeneration medium. The suspension was spread evenly across the medium surface using a sterile spreader. The regeneration medium was incubated under a constant temperature of 25 °C and in a dark environment for several days. When star-shaped colonies could be observed on the surface and the colonies did not touch each other, they were picked out and placed on a new cPDA medium for further cultivation.

### 2.4. Screening and Identification of Integrated Hybrids

Observe the clamp connection of the candidate strains under an optical microscope to verify the affinity and pairing of these two monokaryotic strains [20]. The antagonistic tests and esterase isozyme detection refer to the monokaryotic strain-screening methods. Genomic DNA was extracted using the DP320-02 kit (Tiangen Biochemical Technology Co., Ltd., Beijing, China), and the DNA concentration was determined by 1% agarose gel electrophoresis. ISSR-PCR amplification was performed using the primers and annealing temperatures listed (Table S2) and the PCR reaction conditions listed (Table S3). Based on the ISSR electrophoresis patterns, the genetic similarity coefficients between the strains were calculated using NTSYSpc2.10, and a phylogenetic tree was constructed [21].

### 2.5. Cultivation and Management

Fill the polyethylene mushroom bags with sawdust medium containing 60% moisture and autoclave at 120 °C for 2 h. After cooling, the bags were inoculated with solid spawn of the parental and hybrids, and then cultivated at a constant temperature of 25 °C. After the mycelium become mature, V-shaped incisions were made on the bag surface. Keep the temperature at around 20 °C and control the relative humidity of the air at 85–90%. Harvest when the ear leaves are 80% mature [22]. A total of 14 test strains (12 hybrids and 2 parental strains) were included in this experiment. Each strain was subjected to 15 biological replicates. The experiment was repeated in two independent trials. A total of 420 polyethylene mushroom bags were used in the experiment. Collect data such as the time when the mycelium fills the bag, the time of primordium formation, the harvest, and the yield. Measure the length and width of its ear pieces, as well as its hardness, resilience, cohesiveness and other texture characteristics. The ratio of absorbed water of the fruiting bodies was determined after soaking them at room temperature for 6 h. A colorimeter was used to measure the lightness (ΔL), redness (Δa), yellowness (Δb) and color difference (ΔE) values of the ear pieces.

rehydration ratio = soaked weight/dry weight

$$\Delta E=\sqrt{{\left(\Delta L\right)}^{2}+{\left(\Delta a\right)}^{2}+{\left(\Delta b\right)}^{2}}$$

### 2.6. Transcriptome Analysis

Using the fresh ear pieces of fruiting bodies from the parental strains and hybrid samples, 1 g of each sample was accurately weighed. Each group contained three biological replicates, resulting in a total of nine samples for transcriptome sequencing. All samples were rapidly frozen in liquid nitrogen. The transcriptome sequencing and analysis were conducted by OE Biotech Co., Ltd. (Shanghai, China). Total RNA was extracted using the TRIzol reagent (Invitrogen, CA, USA). RNA integrity was determined using agarose gel electrophoresis. After constructing the complementary DNA (cDNA) library, high-throughput paired-end sequencing (2 × 150 bp) was performed on the IIllumina Novaseq 6000 platform.

The reference genome used in this study was *A. heimuer* version GCA_050574805.1 (JBNRRR000000000, SAMN 44541401) [19]. Raw reads of a fastq format were processed using fastp software (version 0.20.1) and the low-quality reads were removed to obtain the clean reads. Use the HISAT2 software (version 2.1.0) to align the clean reads to the reference genome and calculate gene expression levels (FPKM). All samples generated an average of 24.07 million raw reads, with an average of 23.67 million clean reads after filtering. The average Q30 and GC were 96.72% and 60.55% respectively (Table S4). Differential expression analysis was performed using DESeq2 software (version 1.22.2). Q-value < 0.05 and |log_2_FoldChange| > 1.0 was set as the threshold for significantly differential expression genes (DEGs). The functional enrichment analysis of DEGs was conducted using the GO (Gene Ontology) and KEGG (Kyoto Encyclopedia of Genes and Genomes) databases.

### 2.7. qRT-PCR Verify

To further verify the accuracy of the transcriptome, the adenine phosphoribosyltransferase (*APRT*) gene was selected as the reference gene. This gene has been demonstrated in previous studies to be stably expressed at different developmental stages of fungi and is used for qPCR normalization [23]. Six genes were randomly selected from the transcriptome for quantitative expression analysis. The primers designed based on the transcriptome data are shown (Table S5). The principles of primer design were followed, such as GC content 45–55%, Tm value ranging from 58 °C to 61 °C, and no primer dimer or hairpin structure. The dissolution curve analysis shows that all primers exhibited a single peak. QRT-PCR analysis was performed using the BlazeTaq SYBR Green qPCR Mix 2.0 kit. The specific components and quantities are shown (Table S6). The reaction program was carried out according to the two-step qRT-PCR standard procedure (Table S7). Three biological replicates were set for each experimental group, and each sample was subjected to three technical replicates. Relative gene expression levels were calculated using the 2^−ΔΔCt^ method.

### 2.8. Statistical Analysis

The data were analyzed using SPSS Statistics 24 to perform a one-way analysis of variance (ANOVA), followed by Duncan’s multiple range test (*p* ≤ 0.05). GraphPad Prism software (version 10.1.2) and Adobe Illustrator 2020 were used for chart and figure creation. All data are presented in the form of means ± standard deviation.

## 3. Results

### 3.1. Preparation and Regeneration of Protoplasts

**Figure 1:**
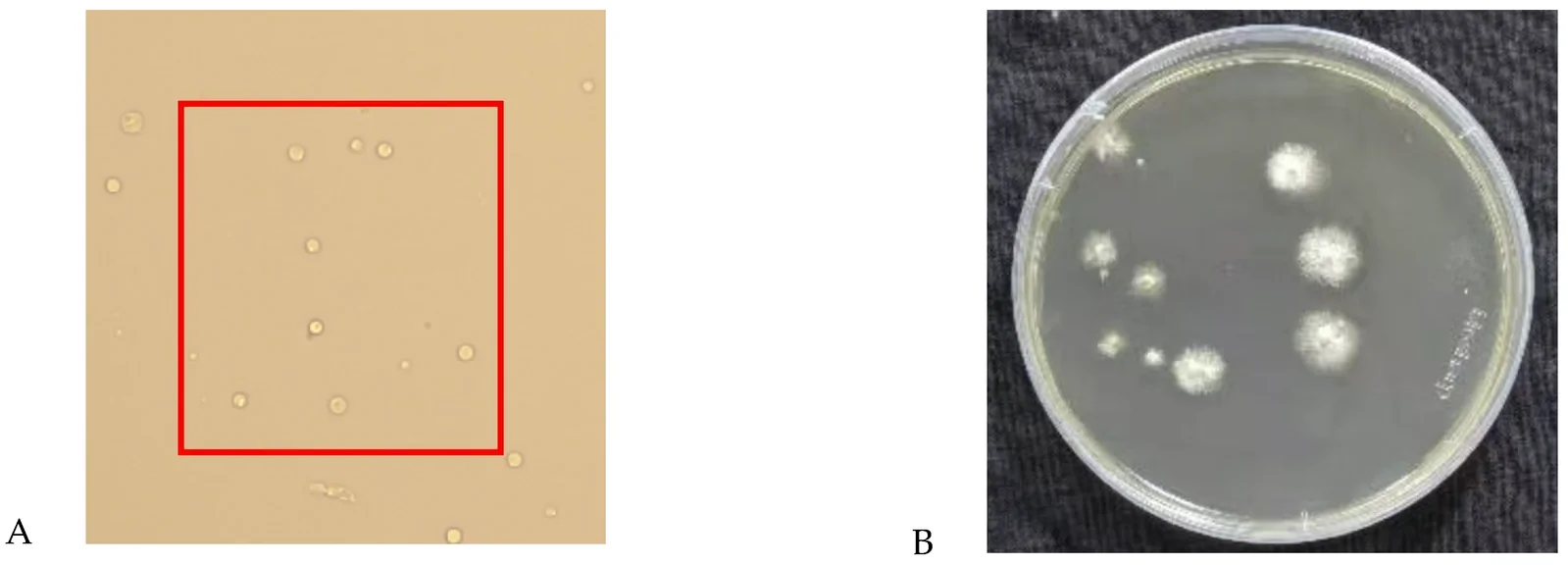
Protoplasts of *A. heimuer* strains. (**A**) Fluorescence micrograph of protoplasts. (**B**) The morphology of protoplasts in the regenerative medium.

The parental protoplasts were obtained through protoplast preparation and regeneration techniques (Figure 1A). The morphology of the protoplasts was observed in the regeneration medium (Figure 1B).

### 3.2. Screening and Identification of Monokaryotic Strain

**Figure 2:**
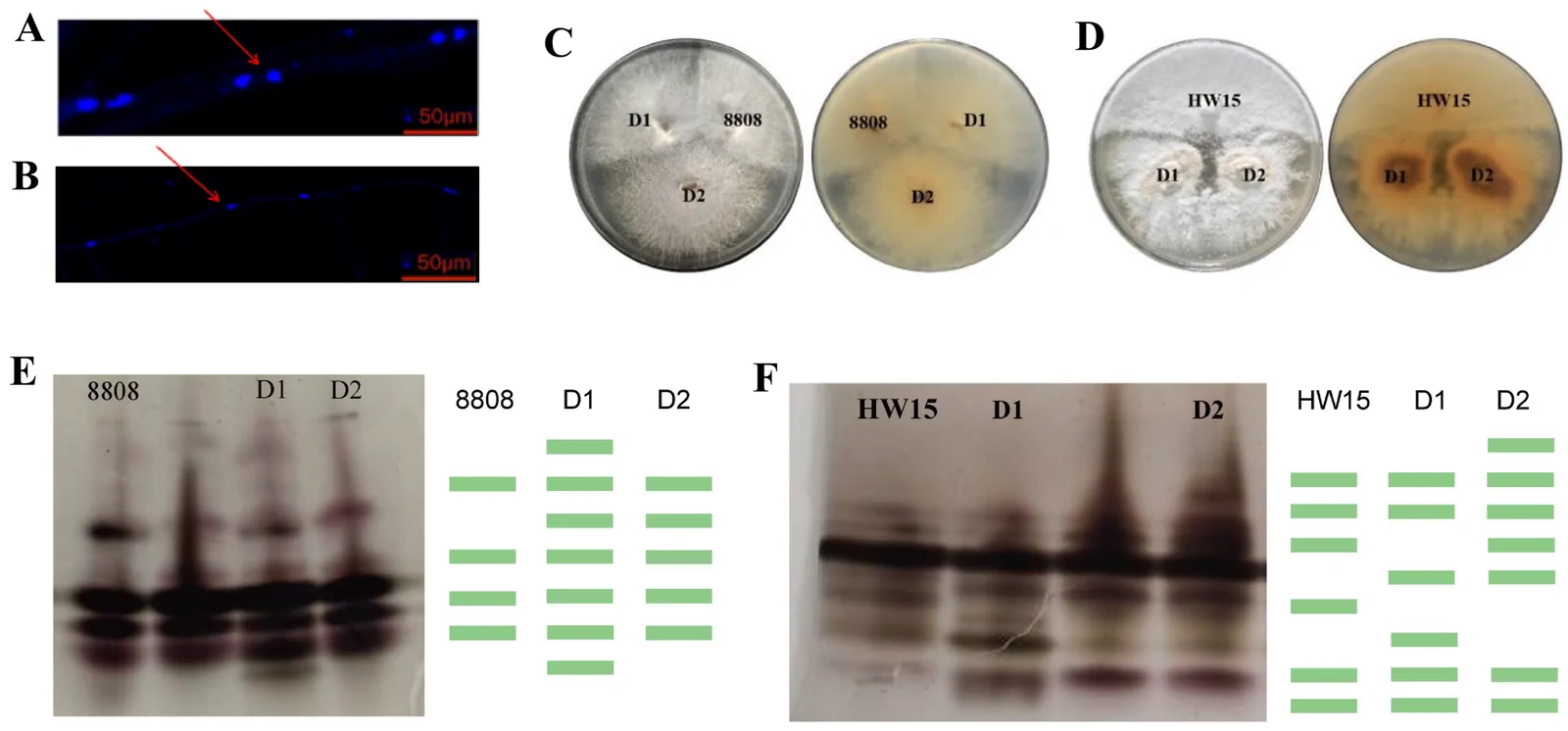
Monokaryotic strain identification. (**A**) Dikaryotic hypha. (**B**) Monokaryotic hypha. (**C**,**D**) Monokaryon–dikaryon antagonism. (**E**,**F**) Esterase isozyme profile.

Observation under a fluorescence microscope: following the regeneration of 8808 and HW15 protoplasts, 200 and 220 test strains were obtained, respectively. When its mycelium is stained with a fluorescent dye, the cell nuclei emit a blue fluorescence. Dikaryotic hypha have two nuclei between their two diaphragms (Figure 2A), whereas monokaryotic hyphae have one nucleus (Figure 2B). After initial screening by fluorescence microscopy, 73 and 100 strains remained from 8808 and HW15 respectively.

Antagonism tests: there is a clear antagonistic line between the same parental and its two monokaryotic strains, while the antagonistic lines between different types of monokaryotic strains of the same parental are not obvious (Figure 2C,D). This indicates that there are significant differences between monokaryotic and dikaryotic strains, whereas the two monokaryotic strains derived from the same parental exhibit a high degree of affinity. Both 8808 and HW15 identified different nuclear types of monokaryotic strains through antagonistic tests and were named 8808-D1 and 8808-D2, HW15-D1 and HW15-D2 respectively.

Esterase isozyme analysis: the 8808 along with 8808-D1 and 8808-D2 detected a total of seven bands. The number of bands for each strain ranged from four to seven, and the common bands among the three strains were four in total (Figure 2E). HW15 along with HW15-D1 and HW15-D2 detected a total of nine bands. The number of bands for each strain ranged from six to seven, and there were five identical bands in total to all three strains (Figure 2F). Compared to their parental strains, monokaryotic strains exhibit specific bands. And the specific bands differ between the two monokaryotic strains. This indicates that although the two monokaryotic strains originate from the same parental strain, differences still exist between them.

The parental strain grows faster than its monokaryotic strains and the mycelium is white and dense. There are some differences in mycelial growth rate and morphology between the two different monokaryotic strains (Table 1).

### 3.3. Preparation, Screening, and Identification of Hybrids

**Figure 3:**
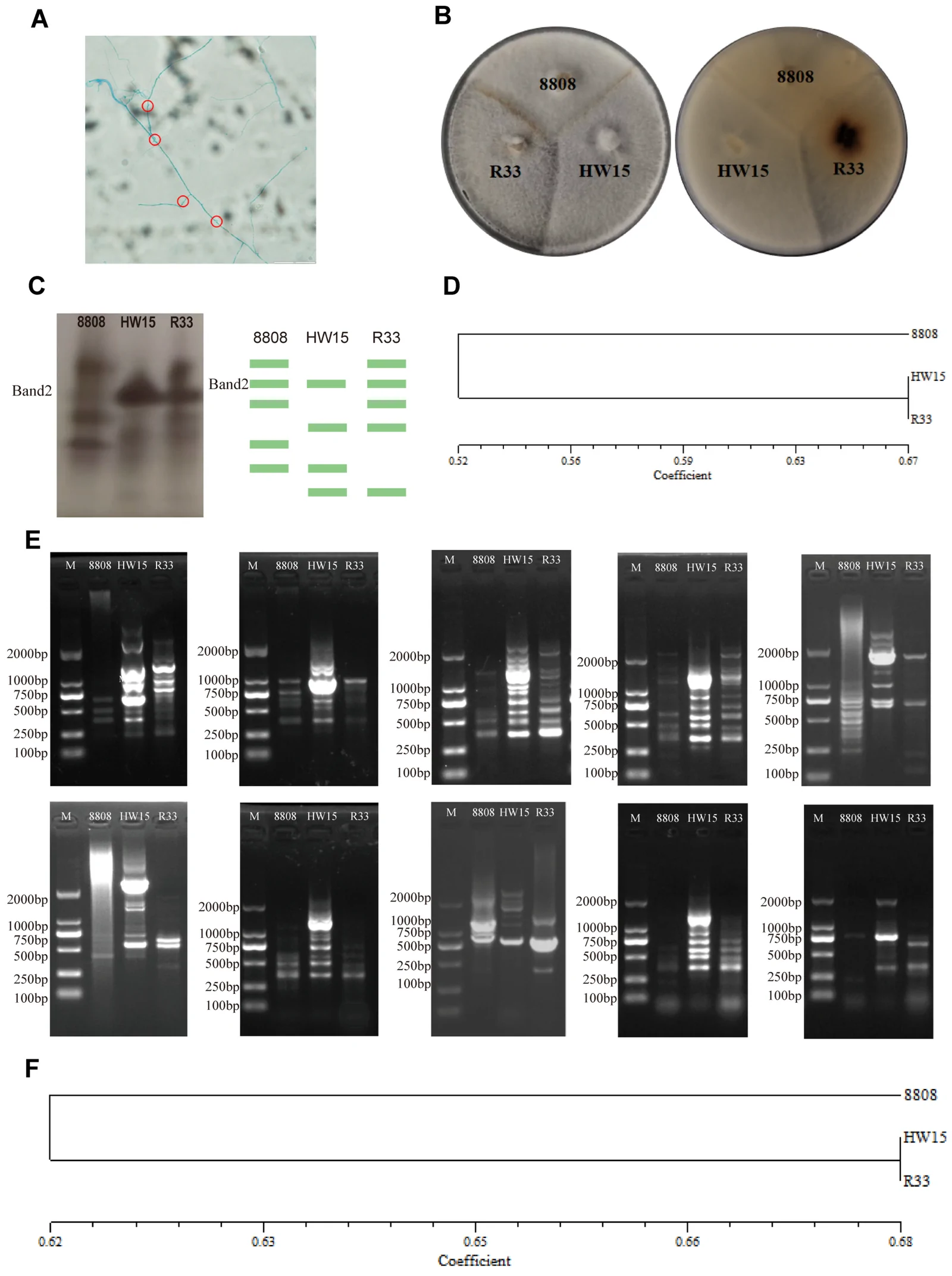
Hybrids identification. (**A**) Clamp connection identification. The area circled in red shows clamp connection. (**B**) Antagonism test between parental strains and hybrid. (**C**,**D**) Cluster analysis of esterase isoenzyme. (**E**) ISSR-PCR product electrophoresis of *A. heimuer* strains. Note: M. DNA marker; the same below. (**F**) Cluster analysis of the genetic relationships of *A. heimuer* strains based on ISSR.

The protoplast suspensions were subjected to heat inactivation and UV inactivation treatments to determine their lethal values. For 8808 and HW15, the lethal values for thermal inactivation were 10 min in a 55 °C water bath and 20 min in a 50 °C water bath, while the lethal values for UV inactivation were 3 min and 5 min respectively. Firstly the parental strain was fused using the PEG-mediated method. A total of 80 strains were obtained. Secondly, after preliminary screening using clamp connection identification, antagonism tests, esterase isozyme analysis and ISSR molecular marker techniques, 12 new strains different from the parental strains were retained. Then, after re-screening by the cultivation, nine strains failed to produce primordia. While three hybrids did. Ultimately, strain R33 with a better growth condition was obtained. The primordia and fruiting bodies of the other two hybrids grew slowly, and the ear pieces of the fruiting bodies remained unexpanded. This indicates that it is a dikaryotic strain resulting from successful fusion (Figure 3A). Antagonism tests were conducted using *A. heimuer* hybrids and their respective parental strains. A distinct antagonism line was observed between the hybrids and parental strains, which allowed for a preliminary determination that the hybrids were different from the parental strains (Figure 3B). A total of seven bands with different migration rates were detected in strains 8808, HW15, and R33. The number of bands for each strain ranged from four to five, and the distribution frequency of band two reached 100.0% (Figure 3C). Cluster analysis of the esterase isoenzyme profiles was performed using NTSYS-PC (2.10e) software. A phylogenetic tree was also constructed. The genetic similarity coefficients among the three strains ranged from 0.52 to 0.67. The similarity coefficient was 0.52 between 8808, HW15 and R33. The similarity coefficient was 0.67 between HW15 and R33 (Figure 3D). The genomic DNA of 8808, HW15 and R33 was subjected to PCR amplification using ISSR primers. The 10 ISSR primers amplified a total of 97 distinct, stable polymorphic fragments of varying lengths. Each primer generated between six and 14 bands in the corresponding genome (Figure 3E). The ISSR band scoring matrix (Table S8) showed that the six and 17 specific bands are inherited by R33 from 8808 and HW15 respectively, while 39 parental-specific bands are lost in R33. The 29 bands are shared by the three strains. And the six bands are specific to R33 and are absent in the parental strains. The similarity levels of the three strains range from 0.62 to 0.68. The similarity coefficient between 8808 and R33 was 0.62. The similarity coefficient between HW15 and R33 was 0.68. This indicates that R33 exhibits significant genetic differences from both parental strains. Therefore, R33 can be considered a new strain distinct from its parental strains (Figure 3F).

**Figure 4:**
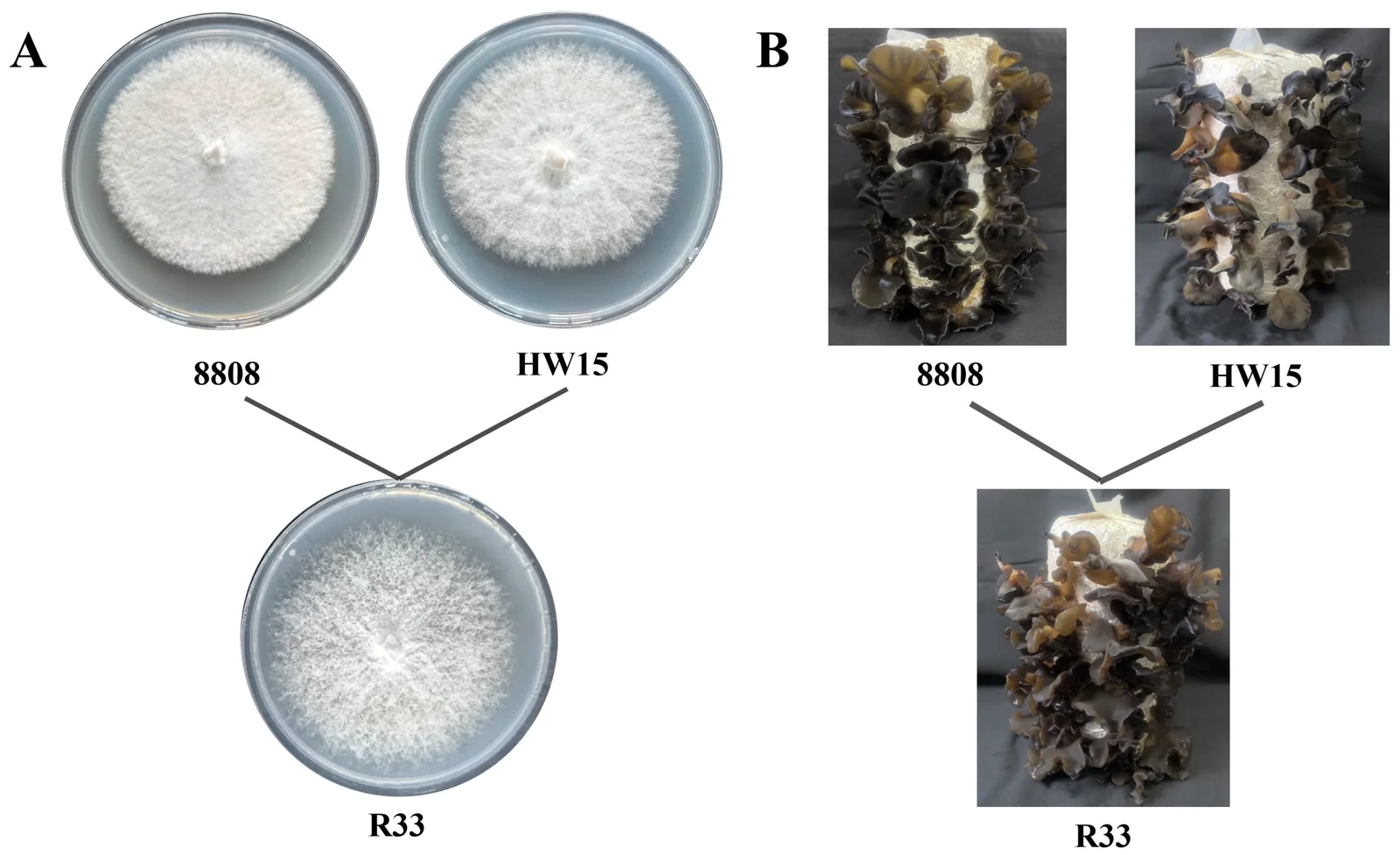
Mycelial and fruiting body morphology of parental strains and hybrid. (**A**) Mycelial morphology of parental strains and hybrid. (**B**) Fruiting body morphology of parental strains and hybrid.

The primordia of the R33 formed 4 days earlier than parental strain HW15, and harvest occurred 6 days earlier. Mycelial growth rate, mycelial colonization time, primordium formation time, and harvest time for R33 all fell between those of 8808 and HW15 (Table 2, Figure 4).

### 3.4. Agronomic Trait Analysis

#### 3.4.1. Texture Profile Analysis (TPA) of Parental Strains and Hybrid

The texture characteristics of the parental strains and hybrid showed significant differences. The ratio of absorbed water of R33 was 9.6% higher than that of its parental HW15 and 29.8% higher than that of 8808. This indicates that the higher the ratio of absorbed water of the fruiting body, the softer its texture, and the lower its hardness, springiness, chewiness, and resilience. The yield of R33 was 14.7% higher than that of its parental HW15, but lower than that of 8808 (Table 3).

#### 3.4.2. Analysis of Ear Pieces Length, Width, and Color Difference

**Figure 5:**
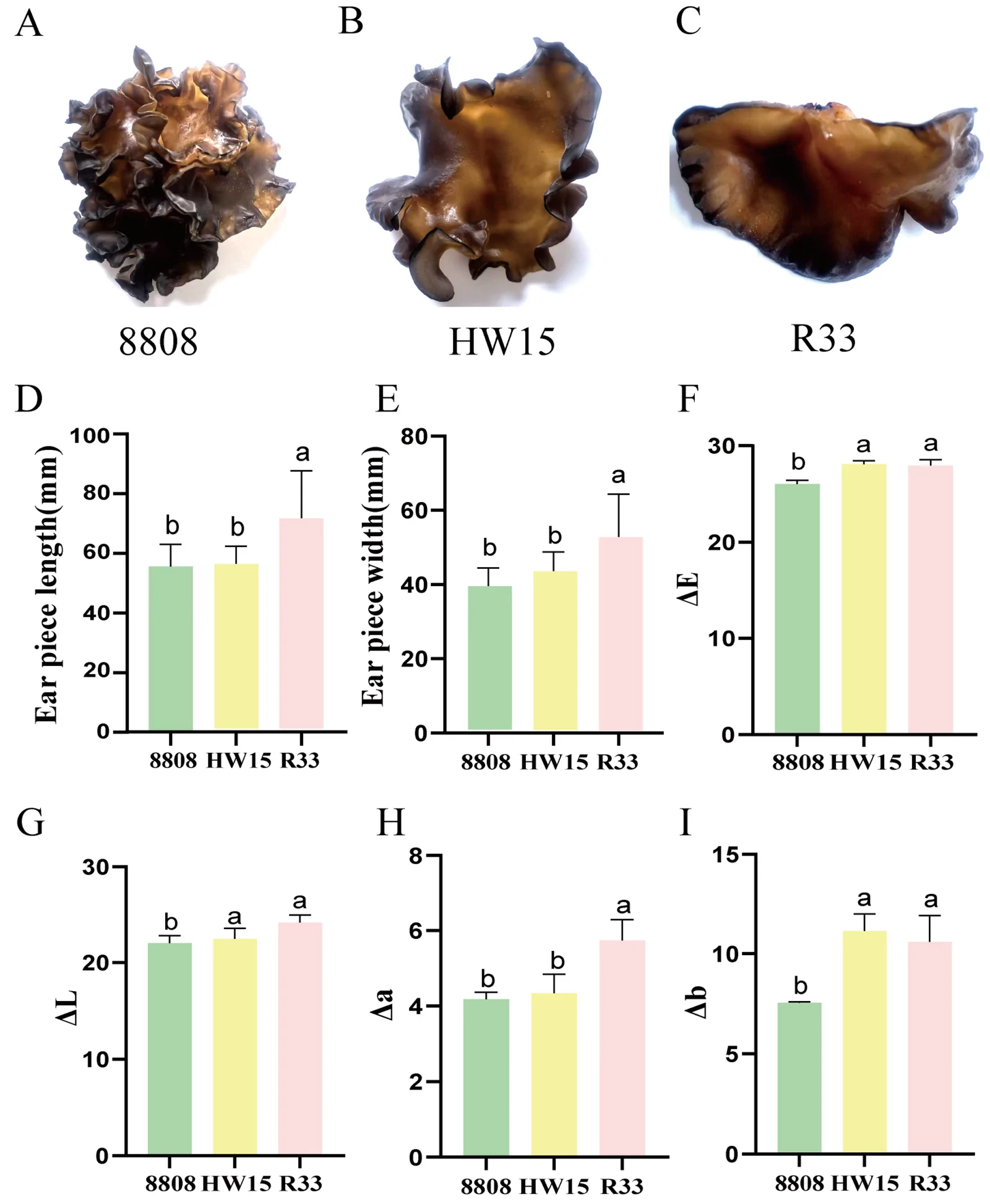
Ear pieces length, width, and color difference in fruiting bodies. (**A**) Fruiting body (ear piece) of the parental strain 8808. (**B**) Fruiting body (ear piece) of the parental strain HW15. (**C**) Fruiting body (ear piece) of the R33. (**D**) Ear piece length. (**E**) Ear piece width. (**F**–**I**) Ear piece color difference in fruiting bodies. Note: Different lowercase letters within the same group indicate a significant difference at *p* < 0.05; while the same letters indicate no significant difference (*p* ≥ 0.05).

The fruiting body shape of 8808 is chrysanthemum-shaped, while the fruiting bodies of HW15 and R33 are single-shaped (Figure 5A–C). The length and width of the ear pieces of R33 are both larger than those of the parental strains (Figure 5D,E). The color difference meter is used to test fresh ears of the fruiting bodies. The ΔE value of HW15 is the highest, indicating the greatest color change. The ΔL value of 8808 is the lowest, and its ear piece color is the darkest. The values of Δa and Δb for all three strains are positive. This suggests that the ear piece color is slightly red and slightly yellow. The Δb value of HW15 is the highest, and its ear piece color is the most yellow (Figure 5F–I).

### 3.5. Transcriptomic Analysis

**Figure 6:**
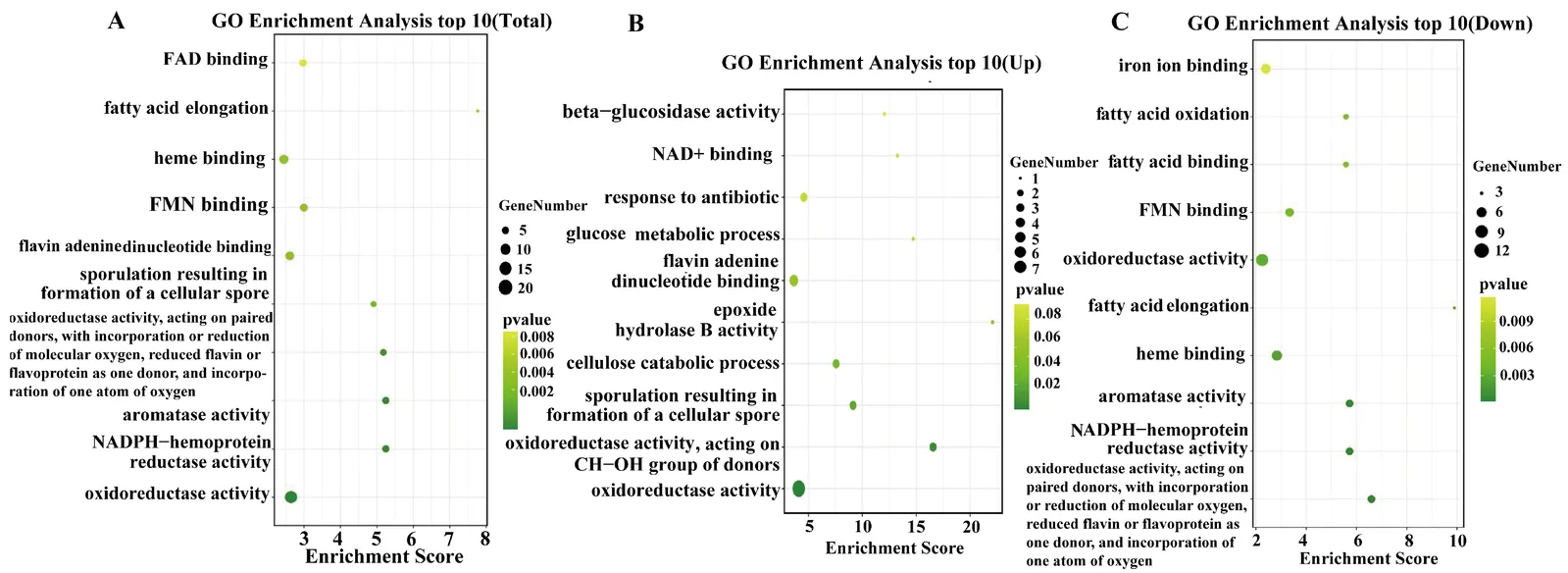
GO enrichment analysis between the parental strains and hybrid. (**A**) GO enrichment analysis top 10 (Total). (**B**) GO enrichment analysis top 10 (Up). (**C**) GO enrichment analysis top 10 (Down). The bubble size represents the number of genes enriched in each pathway, while the color gradient indicates the significance level.

Perform a GO enrichment analysis on the DEGs shared by the parental strains and hybrid. The most significantly enriched DEGs were associated with oxidoreductase activity, fatty acid elongation, and sporulation resulting in the formation of a cellular spore (Figure 6A). Upregulated genes are primarily enriched in oxidoreductase activity and sporulation resulting in formation of a cellular spore (Figure 6B). Downregulated genes are primarily enriched in fatty acid elongation, fatty acid oxidation, and NADPH–hemoprotein reductase activity (Figure 6C).

**Figure 7:**
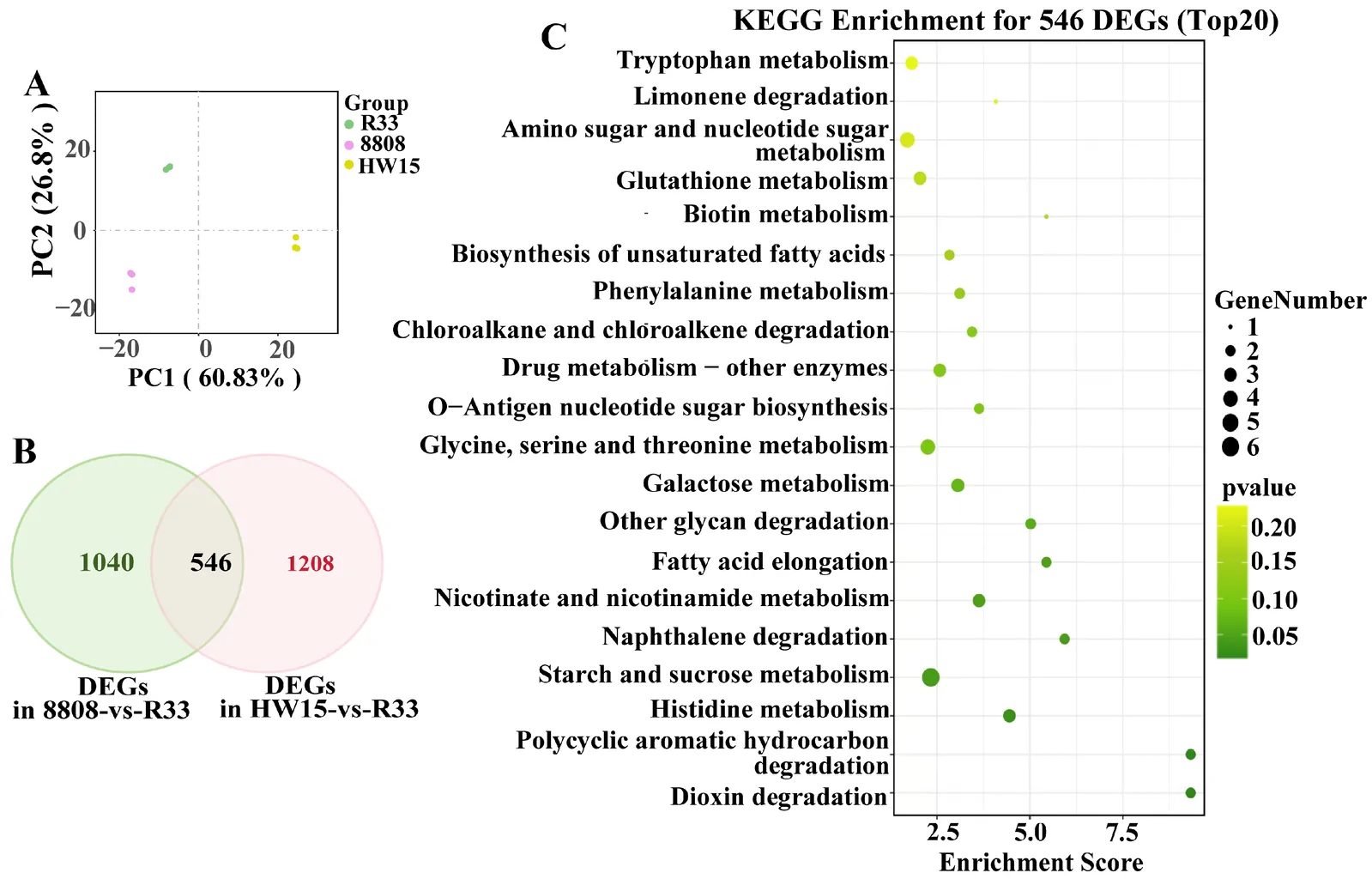
Transcriptome analysis of the parental strains and hybrid. (**A**) Principal Component Analysis of nine samples based on all UniGenes identified by RNA-seq. (**B**) Venn diagram indicates the overlap of DEGs. KEGG enrichment analysis of 546 common DEGs. (**C**) KEGG enrichment for 546 DEGs (Top20).

Principal Component Analysis (PCA) indicates that PC1 accounts for 60.83% of the variability, clearly separating the three groups, and there are extremely small differences among the three biological replicates within each treatment group. This suggests that the transcriptome data is highly reliable (Figure 7A). The Venn diagram analysis of the DEGs showed that 8808-vs-R33 produced 1586 DEGs; HW15-vs-R33 induced 1754 DEGs; and 8808-vs-R33 and HW15-vs-R33 shared 546 DEGs (Figure 7B). Further analysis revealed that the 546 DEGs shared by the two parental strains and the hybrid were primarily enriched in metabolic pathways. They were significantly enriched in starch and sucrose metabolism, amino sugar and nucleotide sugar metabolism, phenylalanine metabolism, glycine, serine and threonine metabolism, histidine metabolism, etc. (Figure 7C).

**Figure 8:**
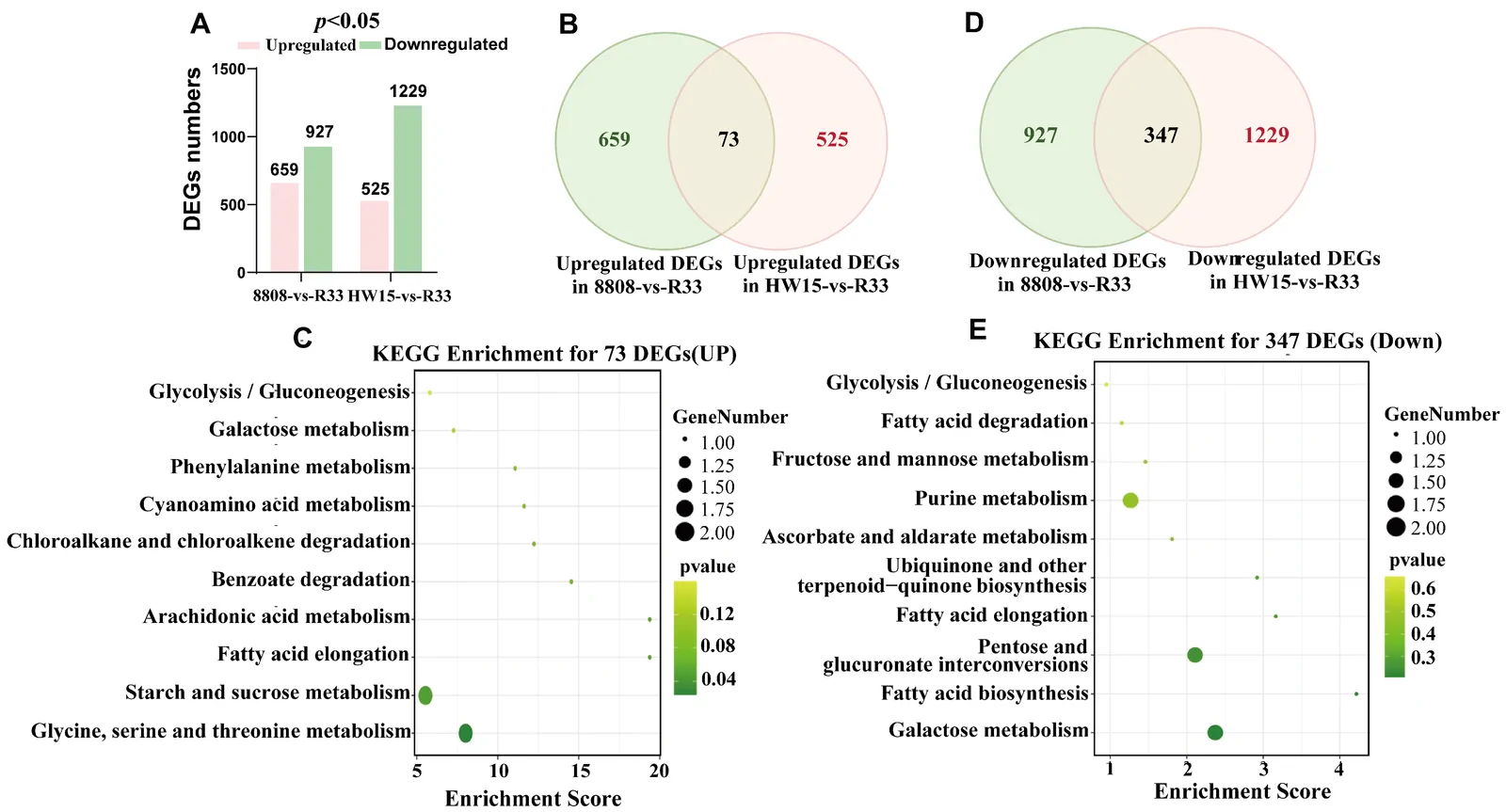
Up- and downregulated genes between the parental strains and the hybrid. (**A**) The number of DEGs in different comparison groups. (**B**) Venn diagram indicates the overlap of DEGs (Up). (**C**) KEGG enrichment for 73 DEGs (Top10). (**D**) Venn diagram indicates the overlap of DEGs (Down). (**E**) KEGG enrichment for 347 DEGs (Top10).

The DEGs identified in the 8808-vs-R33 included 659 upregulated DEGs and 927 downregulated DEGs. The HW15-vs-R33 included 525 upregulated DEGs and 1229 downregulated DEGs (Figure 8A). A total of 73 upregulated DEGs are shared by 8808-vs-R33 and HW15-vs-R33, which are primarily enriched in pathways such as glycine, serine, and threonine metabolism, as well as starch and sucrose metabolism (Figure 8B,C). A total of 347 downregulated DEGs are shared by 8808-vs-R33 and HW15-vs-R33. These downregulated DEGs are primarily enriched in pathways such as purine metabolism, pentose and glucuronate interconversions, and galactose metabolism (Figure 8D,E).

According to previous studies, starch and sucrose metabolism (ko00500) [24], glycine, serine, threonine metabolism (ko00260) [25], benzoate degradation (ko00362) [26], and galactose metabolism (ko00052) [27] are associated with growth and development. Six key genes are identified from the above pathways. The hybrid R33 is designated as the experimental group, and each parental strain (8808 or HW15) served as the corresponding control. Therefore, a positive log_2_FC value indicates higher expression in R33 relative to the parental strain, while a negative log_2_FC value indicates lower expression in R33 relative to the parental strain. In the 8808-vs-R33, gene g10334, g5007, g9525, and g2273 are upregulated by more than two-fold in R33 relative to the parental strain, and genes g1545 and g2094 are significantly downregulated by 51.70% and 52.04% (log_2_FC = −1.05, log_2_FC = −1.06). In the HW15-vs-R33, all six DEGs are upregulated by more than two-fold in R33 relative to the parental strain. Gene g1545 is upregulated by 22-fold, which is the highest among all (Table 4). *CelC*(g10334) and *BglB*(g5007) are upregulated, suggesting that cellulose may be efficiently converted into utilizable glucose in R33 [28,29]. *GalM*(g2273) is upregulated, thereby possibly rapidly catalyzing the conversion of galactose into glucose. Glucose provides sufficient carbon sources and energy for the growth of mycelium and fruiting bodies [30]. *Hbd*(g9525) is upregulated, suggesting a potentially enhanced β-oxidation pathway of fatty acids. This may help maintain intracellular redox homeostasis and provides energy for the formation of fruiting bodies [31].

**Figure 9:**
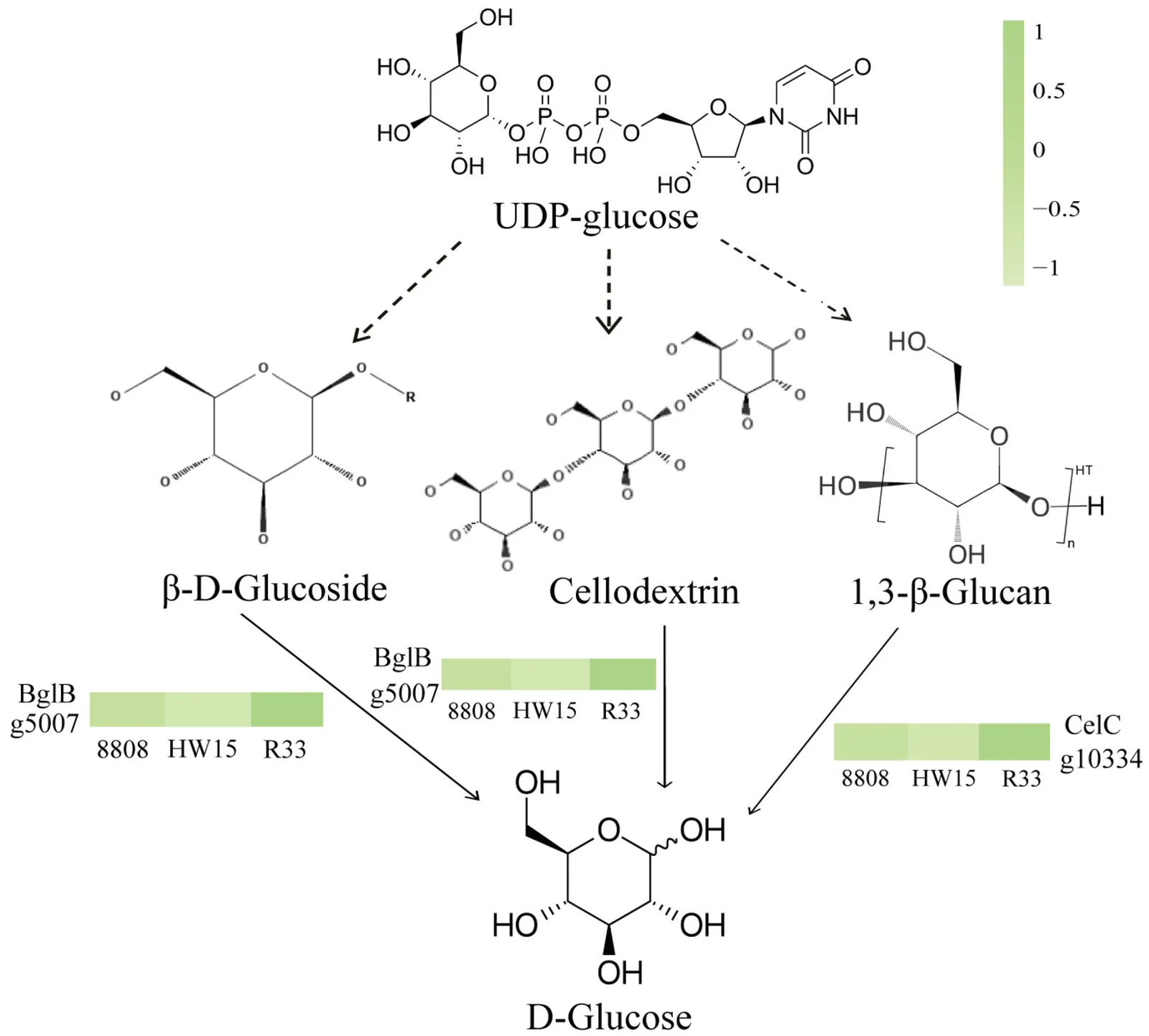
DEGs enriched in the starch and sucrose metabolism pathway. Note: The solid line represents one reaction, the dotted line represents multiple reactions. The color scale denotes absolute |log_2_FC|, where darker shades correspond to larger effect sizes.

The starch and sucrose metabolism may be related to the growth and development of fruiting bodies [32,33]. Analysis of DEGs that are enriched in starch and sucrose metabolic pathways reveals that the gene g10334 encoding glucan 1,3-β-glucosidase (*CelC*) and g5007 which encodes beta-glucosidase (*BglB*) are significantly upregulated in R33, with both showing an increase of more than two-fold. The fold change in gene expression between HW15-vs-R33 is higher than that between 8808-vs-R33. The upregulated expression of *CelC* and *BglB* may promote the hydrolysis of cellobiose, glucan, and glucoside to glucose. This possibly provides an ample supply of carbon and energy for mycelial growth and fruiting body development (Figure 9).

### 3.6. qRT-PCR Verification of DEGs

**Figure 10:**
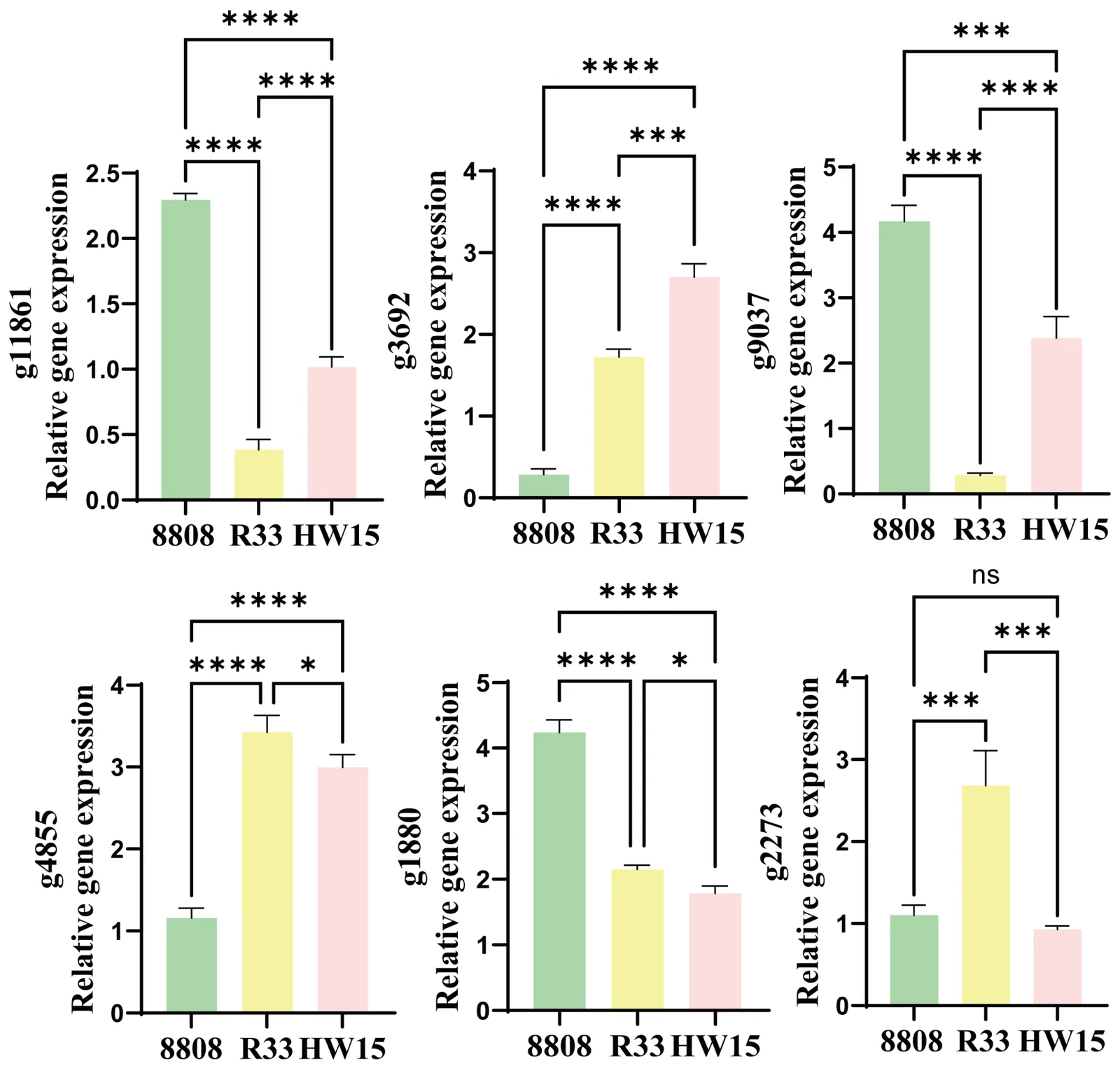
Transcriptome qPCR validation. ns indicates *p* > 0.05 (no statistical significance), * *p* ≤ 0.05, *** *p* ≤ 0.001, **** *p* ≤ 0.0001.

Select the genes with significant differences in log_2_FC values, and use *APRTase* as the internal reference gene for qRT-PCR detection. In R33, genes such as *GSTA*(g11861) and *CelC*(g9037) are downregulated; in contrast, *Egl*(g4855) and *GalM*(g2273) are upregulated. *AMY*(g3692) is upregulated compared to 8808 and downregulated compared to HW15. *TREA*(g1880) is upregulated compared to HW15 and downregulated compared to 8808. The qRT-PCR results showed qualitative agreement with the expression trends observed in the RNA-seq analysis. This indicates that the results of transcriptome data analysis can be applied to subsequent experiments (Figure 10).

## 4. Discussion

Since Yang et al. [34] first reported the interspecific protoplast fusion of *A. heimuer* and *Auricularia polytricha* in 1990, this technology has shown broad application prospects in the improvement of *Auricularia* species. Chen et al. [35] optimized the conditions for preparing and fusing protoplasts within the *A. heimuer* species, increasing the regeneration rate of the fused cells. The interspecific protoplast fusion technology has been further applied to distant hybridization studies of *A. heimuer* and *A. polytricha* [36], and *Auricularia cornea* cv. Yu and *A. heimuer* cv. Bai Muer [13], successfully obtaining fused strains with hybrid advantages. The parental strains of 8808 and HW15 selected for this study are both major cultivated varieties in the Northeast region and represent the characteristics of different varieties. Protoplast fusion technology is used to perform hybridization between these two different types of *A*. *heimuer*. The hybrid is screened using criteria such as shortening growth, increasing yield, and improving the ratio of absorbed water of fruiting bodies. Ultimately, one hybrid that met the objectives is selected. The primordium formation time of the hybrid R33 is 4 days earlier than that of HW15, the harvest time is 6 days earlier, the yield increases by 14.7%, and the ratio of absorbed water increases by 29.8%. This indicates that the hybrid successfully integrated the excellent genetic characteristics of both parental strains. It is a promising candidate strain and exhibits positive hybrid superiority in key productive traits.

A GO enrichment analysis was conducted on the DEGs shared by the parental strain and the hybrid. The results indicate that upregulated DEGs are primarily enriched in oxidoreductase activity and sporulation resulting in formation of a cellular spore. This suggests that the strain may be undergoing a transition from vegetative to reproductive growth, with significant activation of spore formation-related gene expression. Shang et al. [37] and Fu et al. [38] indicated that the activation of reproductive development-related genes is the key to achieving the transformation from mycelium to fruiting body. At the same time, a high-speed metabolism is accompanied by a significant increase in reactive oxygen species production, and the organism ensures the continuity of mycelial extension by enhancing its antioxidant capacity. Hao et al. [39] confirmed in the study of *Hypsizygus marmoreus* that glutathione reductase (GR) regulates mycelial growth and fruiting body development by maintaining cellular redox homeostasis. Silencing of GR leads to an increase in reactive oxygen species accumulation, directly supporting the important role of redox enzyme activity in ensuring the continuity of mycelial extension in edible mushroom development. Chen et al. [40] also found a specific relationship between antioxidant enzyme activity and mycelial growth and fruiting body development.

The DEGs shared by the parental strains and the hybrid are subjected to KEGG enrichment analysis. The upregulated DEGs are mainly enriched in pathways such as glycine, serine and threonine metabolism, as well as starch and sucrose metabolism. This indicates that the amino acid synthesis metabolism may be enhanced during the fruiting body formation stage. At the same time, the decomposition and conversion of starch and sucrose are possibly accelerated. This provides a carbon skeleton and energy supply for rapid cell division and cell wall synthesis. The downregulated DEGs are mainly enriched in pathways such as purine metabolism, pentose and glucuronate interconversions. This suggests a shift in metabolic focus from mycelial vegetative growth to reproductive growth potentially. It is consistent with the metabolic characteristics during the development of various edible mushrooms. In the developmental studies of the *Flammulina velutipes* [41], it was observed that the amino acid metabolism pathway was significantly upregulated during the mature stage of mycelia, promoting the elongation of the mycelial stalk. In *Pleurotus ostreatus* [42], the upregulated expression of amino acid metabolism-related genes was closely related to mycelial growth and fruiting body development. In the study of fruiting body development of *Coprinus comatus* [43], it was found that during the transition from nutritional growth to reproductive growth, amino sugar metabolism was significantly enriched, enhancing cell wall biosynthesis.

The significant upregulated expression of *CelC* and *BglB* indicates that the cellulose-degrading ability of the strain R33 has possibly been enhanced. It may efficiently convert cellulose in the culture medium into readily available glucose [28,29]. This potentially provides a carbon skeleton and energy for mycelial growth and fruiting body development [44]. Shan et al. [45] conducted a study on *A. heimuer* and found that the expression level of cellulase genes was significantly positively correlated with the cellulose degradation amount and yield of the strain. The cellulose degradation rate and yield of the highly expressed strain were significantly higher than those of the lowly expressed strain. Ding et al. [30] also conducted a study on *Volvariella volvacea* and found that the expression of the β-glucosidase gene was closely related to the growth of mycelium on the cellulose substrate. The upregulated expression of *GalM* potentially indicates an enhanced galactose catabolic pathway. This may rapidly catalyze the conversion of galactose into glucose-1-phosphate and UDP-glucose, which enter glycolysis and the tricarboxylic acid (TCA) cycle, providing an ample carbon source and energy for rapid hyphal growth [46]. Seiboth et al. [31] pointed out that the entry of galactose into the central carbon metabolism provides energy and biosynthetic precursors for mycelial growth. *Hbd* is a key enzyme in the fatty acid β-oxidation pathway. Its significant upregulated expression leads to an increase in intermediate products such as acetoacetic acid and acetyl coenzyme A. These enter the TCA cycle to produce a large amount of ATP, accompanied by the generation of reducing power. During the transition of the mycelium to the primordia, it potentially helps maintain intracellular redox homeostasis. It may supply energy and material synthesis guarantees for the formation of the fruiting body tissue [47]. Research by Hou et al. [48] on *Pleurotus ostreatus* demonstrated that the *Hbd*-catalyzed reaction is accompanied by NADH production. This plays a positive regulatory role during the transition from mycelium to primordia, helping to maintain intracellular redox homeostasis. Nagy et al. [49] confirmed that acetyl coenzyme A enters the TCA cycle and generates energy. At the same time, excessive acetyl coenzyme A was also directed towards the biosynthesis of ergosterol and fatty acids to meet the growth requirements of the fruiting body. L-sorbose 1-dehydrogenase is a key enzyme in the L-sorbitol metabolic pathway. In filamentous fungi, the L-sorbitol metabolism may be related to galactose metabolism and cellulase induction. Nogawa et al. [50] found that L-sorbose can directly induce the transcription of cellulase genes. Shida et al. [51] and Adnan et al. [52] further explained that L-sorbose can efficiently induce the expression of cellulase and hemicellulase genes. At the same time, it promoted the conversion of cellulose into glucose, providing carbon sources and energy for the rapid growth of mycelia.

This study successfully obtained a new strain of *A. heimuer* with early maturity and high yield through protoplast fusion. Transcriptome sequencing is used to preliminarily reveal the related metabolic pathways and key genes. However, there are still some limitations. Firstly, the hybrid has only been evaluated in a single environmental condition so far and no multi-region and multi-season demonstration planting has been carried out. Its broad adaptability needs to be verified. Secondly, the genetic stability of the hybrid and the phenotypic stability under different generations have not been systematically evaluated. Whether the early maturity and high yield traits can be stably inherited still needs continuous monitoring.

## 5. Conclusions

Using mycelial protoplast fusion technology, hybrids were generated using the *A. heimuer* 8808 and HW15 as parental strains. Hybrids were screened and identified from multiple perspectives and levels, including morphology, microscopic observation, antagonistic tests, esterase isozyme analysis, and ISSR molecular markers. After repeated screening of the cultivation, a strain R33 with better agronomic traits was identified. The primordium formation time of R33 was 4 days earlier than that of the parental strain HW15, and the harvest time was 6 days earlier. The yield increased by 14.7%, and the ratio of absorbed water increased by 29.8%. Using transcriptomics to identify metabolic pathways that may be associated with early maturity, the genes enriched in the pathways of glycine, serine and threonine metabolism, starch and sucrose metabolism were the most enriched. This is the core pathway by which fungi utilize polysaccharide reserves and regulate intracellular sugar homeostasis. An upregulated expression of the metabolic pathway means that the hybrid can possibly more rapidly break down starch or sucrose in the culture medium into monosaccharides such as glucose, providing a material and energy basis for cell division and hyphal extension.

In conclusion, a promising candidate strain, R33, was selected using protoplast fusion technology. Not only did it achieve synergistic improvements in yield, quality, and growth period, but it also provided a technical model and theoretical reference for hybrid breeding at the protoplast level. Furthermore, by analyzing DEGs in the parental strains and hybrids using transcriptomics, we identified key metabolic pathways related to growth, thereby laying the foundation for molecular breeding in *A. heimuer*.

## Figures and Tables

**Table 1:** Parental and monokaryotic hyphal morphology.

Strains	Mycelial Growth Vigor	Mycelium Color	Average Growth Rate of Mycelium/(cm·d^−1^)
8808	+++	White	0.44 ± 0.02 a
8808-D1	++	White	0.32 ± 0.01 c
8808-D2	+	Yellowish-white	0.31 ± 0.01 c
HW15	++	White	0.40 ± 0.01 b
HW15-D1	+	White	0.29 ± 0.02 c
HW15-D2	+	Yellowish-white	0.31 ± 0.01 c

Note: Different lowercase letters within the same column indicate a significant difference at *p* < 0.05; while the same letters indicate no significant difference (*p* ≥ 0.05). +: fair; ++: dense; +++: luxuriant.

**Table 2:** Mycelial growth rate and morphology of parental strains and hybrid.

Strain	8808	HW15	R33
Mycelial growth vigor	+++	+++	++
Mycelium color	White	White	Yellowish-white
Average growth rate of mycelium/(cm·d^−1^)	0.82 ± 0.019 a	0.77 ± 0.01 b	0.79 ± 0.02 ab
Colony margin morphology	Regular margin	Regular margin	Irregular margin
Aerial mycelium distribution	Uniform and robust	Relatively uniform and robust	Relatively uniform and relatively robust
Mycelial colonization time (d)	45.00 ± 0.89 b	48.17 ± 1.47 a	46.33 ± 1.03 a
Primordium formation time (d)	52.83 ± 1.83 b	58.20 ± 1.15 a	54.00 ± 0.58 b
Harvest time (d)	79.60 ± 0.49 b	87.17 ± 1.26 a	81.33 ± 1.43 a

Note: Different lowercase letters within the same row indicate a significant difference at *p* < 0.05; while the same letters indicate no significant difference (*p* ≥ 0.05). ++: dense;+++: luxuriant. Data were compared horizontally across the table.

**Table 3:** Yield, the ratio of absorbed water and texture profile analysis (TPA) of parental strains and hybrid.

Strains	8808	HW15	R33
Hardness (N)	4321.74 ± 369.49 a	2584.85 ± 254.64 bc	2303.59 ± 170.09 c
Springiness (mm)	0.77 ± 0.01 a	0.73 ± 0.21 a	0.68 ± 0.04a
Cohesiveness	0.49 ± 0.05 a	0.27 ± 0.036 b	0.33 ± 0.03 b
Gumminess (N)	2107.03 ± 336.02 a	700.91 ± 148.57 c	715.51 ± 95.35 c
Chewiness (mj)	1625.42 ± 260.53 a	512.29 ± 123.25 b	492.42 ± 89.89 b
Resilience	0.39 ± 0.05 a	0.18 ± 0.03 b	0.16 ± 0.02 b
Rehydration rate	1: (11.21 ± 0.81 b)	1: (13.26 ± 0.80 a)	1: (14.54 ± 0.48 a)
Average yield per bag (g)	8.27 ± 1.96 a	6.52 ± 0.64 b	7.48 ± 1.251 a

Note: Different lowercase letters within the same row indicate a significant difference at *p* < 0.05; while the same letters indicate no significant difference (*p* ≥ 0.05).

**Table 4:** Significant DEGs.

Pathway	Gene ID	Gene Symbol	Description	8808-vs-R33		HW15-vs-R33	
				Log_2_FC	Regulation	Log_2_FC	Regulation
Starch and sucrose metabolism (ko00500)	g10334	*CelC*	Endoglucanase C	1.12	Up	1.38	Up
	g5007	*BglB*	Thermostable beta-glucosidase B	1.17	Up	1.60	Up
Glycine, serine and threonine metabolism (ko00260)	g1545		L-sorbose 1-dehydrogenase	−1.05	Down	4.50	Up
	g2094		L-sorbose 1-dehydrogenase	−1.06	Down	2.21	Up
Benzoate degradation (ko00362)	g9525	*Hbd*	3-hydroxybutyryl-CoA dehydrogenase	1.63	Up	1.10	Up
Galactose metabolism (ko00052)	g2273	*GalM*	Aldose 1-epimerase	1.20	Up	1.23	Up

## Data Availability

The data presented in this study are openly available in NCBI at https://www.ncbi.nlm.nih.gov/bioproject/ (accessed on 22 July 2026), reference number PRJNA1475071.

## Supplementary Materials

The following supporting information can be downloaded at: https://www.mdpi.com/article/10.3390/jof12080591/s1, Table S1. Preparation of the main mediums. Table S2. 10 ISSR primer sequences and annealing temperature. Table S3. Component and volume of PCR system. Table S4. List of sequencing data quality preprocessing results Table S5. qRT-PCR validation primers for transcriptome data. Table S6. qRT-PCR system. Table S7. qRT-PCR amplification procedures. Table S8. ISSR band-scoring table. Figure S1: The colonies on the regeneration medium.

## Abbreviations

The following abbreviations are used in this manuscript:

*A*. *heimuer*	*Auricularia heimuer*
DEGs	Differentially Expressed Genes
ISSR	Inter-Simple Sequence Repeat
cPDA	complete Potato Dextrose Agar
