# Supplementary material for: Breeding of Early Maturity, High-Quality Auricularia heimuer Strains Using Protoplast Fusion
Source: J Fungi (Basel). 2026 Aug 10;12(8):591. doi: 10.3390/jof12080591 (PMC13514120; doi:10.3390/jof12080591)
Supplement: Supplementary file 1 [file jof-12-00591-s001.zip › Supplementary file.pdf]

Tables S1 Preparation of the main mediums

Medium	Composition
cPDA medium	Potato 200.0g(boiled and filtered), Glucose 20.0g, $MgSO_4$1.5g, $KH_2PO_4$3.0g, Peptone 0.5g, Agar14.0g, dissolved in distilled water to a final volume of 1000mL.
Liquid regeneration medium	Potato 200.0g(boiled and filtered), Mannitol 109.3 g, Glucose 20.0g, Peptone 0.5g, $KH_2PO_4$3.0g, Yeast extract 2.0g, $MgSO_4$ 1.5g, dissolved in distilled water to a final volume of 1000mL.
CYM medium	Potato 200.0g(boiled and filtered), Glucose 20.0g, $KH_2PO_4$3.0g, Peptone 0.5g, Yeast extract 2.0g,$MgSO_4$1.5g, Agar20.0g, dissolved in distilled water to a final volume of 1000mL.
Sucrose hyperosmotic regeneration medium	CYM medium supplemented with 171.0g Sucrose, dissolved in distilled water to a final volume of 1000mL.
Sawdust medium	Sawdust 830g, wheat bran 150g, lime 10 g, gypsum 10g, mixed to a total weight of 1000g.

Table S2 10 ISSR primer sequences and annealing temperature

Primer number	Primer sequence	Annealing temperature/°C
1	AGAGAGAGAGAGAGAGC	50
2	AGAGAGAGAGAGAGAGG	52
3	GAGAGAGAGAGAGAGAC	47
4	GAGAGAGAGAGAGAGAAC	48
5	TGCACACACACACAC	50
6	CGTGTGTGTGTGTGT	50
7	GGAGTGGTGGTGGTG	52
8	GTGACGACTCTCTCTCTCT	53
9	CCAGTGGTGGTGGTG	53
10	CACCACACACACACACA	53

Table S3 Component and volume of PCR system

Component	Volume
2×*Taq* PCR Mix	10
Primer	1
DNA template	2
ddH_2O	7

Table S4 List of sequencing data quality preprocessing results

Sample	Raw Reads(M)	Raw Bases(G)	Clean Reads(M)	Clean Bases(G)	Valid Bases(%)	Q30 (%)	GC (%)
8808-1	24.26	7.15	23.83	7.03	98.25	96.70	60.67
8808-2	24.12	7.14	23.77	7.03	98.54	96.81	60.62
8808-3	24.08	7.11	23.68	6.99	98.32	96.79	60.45
HW15-1	24.25	7.14	23.79	7.01	98.12	96.78	60.09
HW15-2	23.86	7.06	23.51	6.95	98.53	96.59	60.43
HW15-3	23.80	7.02	23.39	6.90	98.26	96.86	60.12
R33-1	24.04	7.07	23.56	6.93	98.00	96.51	60.70
R33-2	24.01	7.10	23.65	6.99	98.49	96.72	60.98
R33-3	24.18	7.16	23.86	7.06	98.65	96.71	60.89

Table S5 qRT-PCR validation primers for transcriptome data

Gene ID	Forward primer（5'-3'）	Reverse primer（5'-3'）
APRTase	CGCTCGCAGGGTCTCAAGAATG	GCCGTGGAAGTTCGTCAAGCA
g12404	GACGACTGACACTGGAAACC	TCTTCGACCTGGATCTCATG
g2273	GTGGGATATGCCATGGAGAT	GCAACTGCCAACGGTAAAC
g2276	CGCCGCTGACGAGAATAAT	CGAAGAGTGCTTGACGGTATG
g2656	TCATCGGAGTCAACACCTACG	TTATCGCATTGCCCGTCT
g3662	GCTTTTCGTTGGCTTTCTC	TTCGGGATGGATGATGTTG
g3877	CCCCGTTGATAGACGAAGGT	TAGGGAATGGGAGTGGAGGT
g5908	GCTATGGCGAATATCAACCG	TGAGCATCGTGGAAAGAAGG
g6499	CATCAACAACAACTGGGACTTC	TGGATACCCTGCGGACTAAC
g7634	CTTCGGAAAACCTGGAGACC	TGAAAGCGTAACGTGAGCC
g7671	CAAATGCTTAACGCGGACC	CCTCGGCGAAATAGACAGAC
g7797	TCAAAACTGGGGCCTGTAC	CCTGGTCCGAGATGATGTAGT
g7811	TAGCGTCAAGAAGCCTGTAGC	TCATCAGTATTCGCAACCAGG

Table S6 qRT-PCR system

Component	Volume
5x Blaze Taq qPCR Mix	4μL
PCR forward primer (2 μM)	2pL
PCR reverse primer (2 μM)	2μL

Template	2μL
ROX Reference Dye(30μM）	0.1μL
Using ROX Reference Dye	9.9μL
Total	20uL

Table S7 qRT-PCR amplification procedures

Cycles	Step	Temperature(℃)	Time(s)	Detection
1	Pre-denaturation	95	30	No
40	Denaturation	95	10	No
	Annealing and extension	60	30	Yes

Table S8 ISSR band-scoring table

		8808	HW15	R33	Inheritance description
Primers 1	1	0	1	0	Lost
	2	0	0	1	Lost
	3	0	1	1	HW15
	4	0	1	0	Lost
	5	1	1	1	Shared band
	6	0	1	1	HW15
	7	1	1	1	Shared band
	8	0	1	1	HW15
	9	1	1	1	Shared band
	10	1	1	1	Shared band
	11	1	1	1	Shared band
Primers 2	12	0	0	1	Specific
	13	1	1	1	Shared band
	14	1	0	1	8808
	15	0	0	1	Specific
	16	1	1	1	Shared band
	17	1	1	1	Shared band
	18	0	1	1	HW15
	19	1	1	1	Shared band
	20	1	1	1	Shared band
	21	1	1	1	Shared band
	22	0	1	0	Lost
Primers 3	23	0	1	0	Lost
	24	0	1	0	Lost
	25	1	1	0	Lost
	26	0	1	1	HW15
	27	1	1	1	Shared band
	28	1	1	1	Shared band
	29	1	1	1	Shared band
Primers 4	30	0	1	1	HW15

	31	0	1	1	HW15
	32	0	1	1	HW15
	33	1	1	1	Shared band
	34	1	1	1	Shared band
	35	1	1	1	Shared band
	36	0	1	0	Lost
	37	1	0	1	8808
Primers 5	38	0	1	0	Lost
	39	1	1	1	Shared band
	40	0	1	1	HW15
	41	0	1	0	Lost
	42	0	1	1	HW15
	43	1	0	0	Lost
Primers 6	44	0	1	0	Lost
	45	0	1	0	Lost
	46	0	1	1	HW15
	47	0	1	1	HW15
	48	1	1	0	Lost
	49	1	0	0	Lost
	50	1	0	0	Lost
	51	1	1	1	Shared band
	52	1	1	0	Lost
	53	1	0	1	8808
	54	1	0	0	Lost
	55	1	0	0	Lost
	56	1	0	0	Lost
	57	1	0	1	8808
Primers 7	58	0	1	0	Lost
	59	0	1	0	Lost
	60	1	1	0	Lost
	61	0	1	0	Lost
	62	0	1	0	Lost
	63	0	1	0	Lost
	64	1	1	0	Lost
	65	0	1	1	HW15
	66	1	1	1	Shared band
	67	1	1	0	Lost
Primers 8	68	0	1	0	Lost
	69	1	1	0	Lost
	70	0	1	0	Lost
	71	1	1	0	Lost
	72	1	1	1	Shared band
	73	1	0	0	Lost
	74	1	1	1	Shared band

	75	1	0	1	8808
Primers 9	76	0	1	0	Lost
	77	0	1	1	HW15
	78	0	0	1	Specific
	79	0	1	1	HW15
	80	0	0	1	Specific
	81	0	0	1	Specific
	82	0	1	1	HW15
	83	0	1	1	HW15
	84	0	0	1	Specific
	85	1	1	1	Shared band
	86	1	1	1	Shared band
	87	1	1	1	Shared band
	88	0	1	0	Lost
	89	1	1	1	Shared band
Primers 10	90	0	1	0	Lost
	91	0	1	0	Lost
	92	0	1	0	Lost
	93	1	1	1	Shared band
	94	1	0	1	8808
	95	1	1	1	Shared band
	96	1	1	0	Lost
	97	1	1	1	Shared band

Notes: 1.Shared band: It indicates that the bands are shared by 8808, HW15 and R33. 2.Lost: It indicates that the R33 is not inherited from the parental strains. 3.Specific: The band is specific to R33 and is absent in the parental strains.

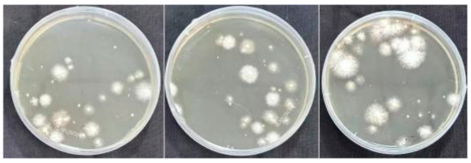

Figure S1: The colonies on the regeneration medium
